# 

**Published:** 1864-12

**Authors:** 


### DENTAL REGISTER

### OF THE WEST.

EDITED BY

J. TAFT AND GEO. WATT.

DECEMBER,–1864.

---

**MONTHLY:**

**Three Dollars per Annum, in advance.**

---

CINCINNATI:
J. TAFT, PUBLISHER.

J. Taft, Dentist, 172 Race Street.

